# Usefulness of Early Postoperative Atrial Fibrillation Burden as a Predictor of Late Recurrence after Maze Procedures

**DOI:** 10.1186/1749-8090-10-S1-A329

**Published:** 2015-12-16

**Authors:** Yeong Jeong Jeon, Dong Seop Jeong, Pyo Won Park, Young Tak Lee

**Affiliations:** 1 Department of Thoracic and Cardiovascular Surgery, Samsung Medical Center, Sungkyunkwan University School of Medicine, Seoul, 135-710, Korea

## Purpose

It is difficult to evaluate the efficacy of maze procedures because a true atrial fibrillation (AF) burden measurement during follow-up is not yet clinically available. The aim of this study is to evaluate the usefulness of early postoperative AF burden as a predictor of late AF recurrence after the maze procedure.

## Methods

Between January 2000 and November 2009, we enrolled 508 consecutive patients (284 females, aged 55 ± 12 years) who underwent the maze procedure with other cardiac operations. The early postoperative AF burden was measured by continuous monitoring of the cardiac rhythm during hospitalization. The post procedural rhythm was checked with a serial electrocardiogram every year. The mean follow up duration was 58 ± 32 (maximum, 10.4 years) months.

## Results

**Figure 1:**
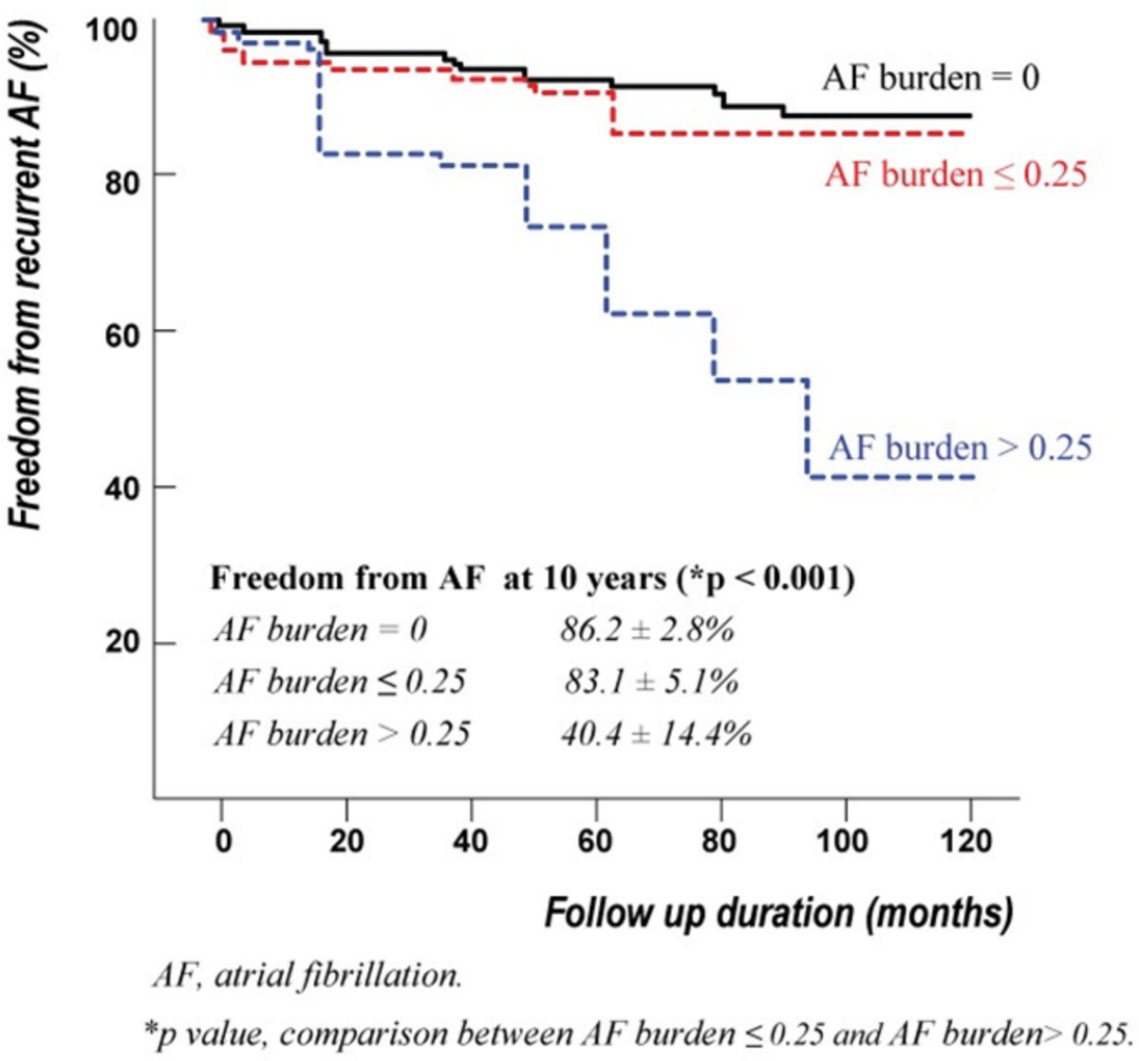
Freedom from late recurrence of atrial fibrillation

The hospital mortality was 0.2% (1/508). Late cardiac-related deaths occurred in 25 patients (4.9%). 419 patients (86.9%) remained AF-free at the end of the follow-up period. The early postoperative AF burden was correlated with age, AF duration, atrial F wave, and left atrial volume index (Table 1). On Cox regression analysis, independent predictors of AF recurrence were AF duration (p < 0.001) and early postoperative AF burden (p < 0.001). On receiver operating characteristic curve analysis, we found that early postoperative AF burden ≥ 0.25 predicted AF-free survival with a sensitivity of 77% and a specificity of 62% (area under the curve (AUC) = 0.768) and AF duration ≥ 30 months predicted AF-free survival with a sensitivity of 70% and a specificity of 60% (AUC = 0.729). Freedom from AF recurrence at 10 years was significantly lower in patients with early postoperative AF burden > 0.25 (Figure 1).

**Table 1:** Variables correlated with early postoperative atrial fibrillation burden

Variables	γ	P value
Age (year)	0.234	<0.001
Atrial fibrillation duration (month)	0.291	<0.001
Atrial fine F wave (<1 mm)	0.263	<0.001
Left atrial volume index (ml)	0.174	0.002

γ, Pearson correlation.

## Conclusions

Measurement of the early postoperative AF burden was helpful in predicting AF recurrence. We suggest that greater efforts to prevent AF recurrence should be made in patients with high early postoperative AF burden and long AF duration.

